# Molecular epidemiology of hepatitis A outbreaks and sporadic cases, Latvia, 2017 to 2019

**DOI:** 10.2807/1560-7917.ES.2022.27.11.2100415

**Published:** 2022-03-17

**Authors:** Oksana Savicka, Reinis Zeltmatis, Jelena Storozenko

**Affiliations:** 1 Riga East Clinical University Hospital, Infectology Centre of Latvia, National Microbiology Reference Laboratory, Riga, Latvia

**Keywords:** hepatitis A, genotype, MSM, outbreak, phylogenetic analysis, Latvia

## Abstract

**Background:**

Hepatitis A is an acute infection of the liver caused by hepatitis A virus (HAV). Molecular detection and typing of the HAV VP1/P2A genomic region is used for genotyping and outbreak investigations. After a large hepatitis A outbreak in Latvia in 2007–08, only sporadic cases were registered until 2017 when a rise in cases occurred. During 2017–19, 179 laboratory-confirmed hepatitis A cases were notified in Latvia.

**Aim:**

To investigate the observed increase in hepatitis A cases during 2017 and to determine whether these cases were linked to one another, to risk groups, or to other outbreaks. The majority of HAV samples (69.8%) were typed.

**Methods:**

The VP1/P2A genomic region of HAV was amplified and sequenced for 125 case serum samples. Information about hepatitis-related symptoms, hospitalisation, vaccination, a possible source of infection and suspected countries of origin of the virus were analysed for sequenced cases.

**Results:**

Most HAV strains were subgenotype IA (n = 77), of which 41 were strains circulating among men who have sex with men (MSM) populations in Europe (VRD_521_2016 (n = 32), RIVM-HAV16–090 (n = 7) or V16–25801 (n = 2)). Forty-four cases were subgenotype IB and four cases subgenotype IIIA. However, other clusters and sporadic cases were detected with or without identifying the epidemiological link.

**Conclusion:**

This work represents molecular epidemiological data of hepatitis A cases in Latvia from 2017 to 2019. Molecular typing methods allow identification of clusters for public health needs and establishing links with other outbreaks, and to compare Latvian strains with reported strains from other countries.

## Introduction

Hepatitis A is a viral liver disease that can cause mild to severe illness. According to the World Health Organization (WHO), hepatitis A affects 1.5 million people worldwide annually, although true incidence of infection could be 10 times higher. In 2016, WHO estimated that hepatitis A accounted for 0.5% of mortality from all forms of viral hepatitis [1]. The disease is caused by the hepatitis A virus (HAV), a *Hepatovirus* in the family *Picornaviridae,* which is transmitted primarily by exposure to contaminated food or water, or through exposure to infected individuals [2,3]. In countries where the risk of infection from food or water is low, outbreaks disproportionately affect risk groups including men who have sex with men (MSM) and people who inject drugs (PWID) [4].

In Latvia, hepatitis A is a notifiable disease, with all cases reported to the Centre for Disease Prevention and Control of Latvia (CDC) [5]. Since the last HAV outbreak in Latvia, which occurred in 2008–09 and resulted in 5,107 cases, the number of hepatitis A cases has remained steady at around 10 cases per year, all of which have been sporadic and without epidemiological links to each other. However, in 2017, an increase in cases was observed, followed by continued case reporting up to 2019.

During hepatitis A outbreak investigations, molecular detection of the VP1/P2A genomic region of HAV is used for genotyping to differentiate HAV strains based on nucleotide sequence analyses and thereby to understand links between strains [6,7]. Sequence variation within the VP1/P2A junction is used to define genotypes and subgenotypes, which have 15% and 7–7.5% nucleotide (nt) variations between isolates, respectively [8]. Four of the seven HAV genotypes identified (I, II, III and VII) are of human origin, of which genotypes I, II, III are divided into two subgenotypes (A and B) [9,10]. Nucleotide sequence data indicate that HAV genotype I is the most prevalent worldwide, with subgenotype IA more common than IB.

The aim of this study was to investigate the reason for the observed increase in cases of hepatitis A in the middle of 2017. As cases continued to be reported in 2018–19, the majority of HAV samples were typed to determine whether these cases were linked to one another, to risk groups or connected other to outbreaks. We describe our investigations, including genotype sequence characteristics of the cases and phylogenetic analyses, and summarise the available surveillance data of HAV in Latvia from 2017–19.

## Methods

### Study setting

Riga East Clinical University Hospital, Infectology Centre of Latvia is the main institution in the field of infectious diseases and houses Latvia’s National Microbiology Reference Laboratory (NRL). The NRL is Latvia’s primary centre of detection, confirmation and molecular typing of HAV. Clinicians should notify probable and confirmed cases, and laboratories are required to report positive HAV results to the CDC. Laboratory diagnosis is based on at least one of the following three laboratory criteria according to the 2018 EU case definition for acute hepatitis A: (i) detection of hepatitis A virus nucleic acid in serum or stool, (ii) hepatitis A virus-specific antibody response or (iii) detection of hepatitis A virus antigen in stool [11]. Serological tests for anti-HAV IgM are the most common and a mainstay in the diagnosis. When the CDC receives notification reports from clinicians or laboratories, all cases of hepatitis A are investigated by the in-house epidemiologists, who contact the HAV patient and collect information about e.g. hospitalisation, vaccination, hepatitis symptoms, and travel during the incubation period.

### Data collection

We examined all laboratory-confirmed hepatitis A cases (n = 179) were notified in Latvia between 2017 and 2019 [12-14]. The serum samples collected by the NRL (125/179) were genotyped and tested for the presence of anti-HAV IgM antibodies by ELISA using Elecsys anti-HAV IgM (Roche Diagnostic, Mannheim, Germany) or Architect HAVAb IgM (Abbott, Wiesbaden, Germany). Serum samples collected by private diagnostic laboratories (n = 54) were not retained for further analysis.

We collected demographical (age, sex, country of birth, country of residence) and epidemiological data including (i) hepatitis-related symptoms (fatigue, abdominal pain, loss of appetite, intermittent nausea and vomiting, and at least one of the following three: fever, jaundice, elevated serum aminotransferase levels), (ii) information about hospitalisation, (iii) vaccination against HAV, (iv) possible sources of infection (contact with an HAV case, travel to an endemic country, MSM, PWID, food-borne or unknown) and (v) suspected country of origin of the virus for typed HAV samples.

### Data analysis

To describe the demographic characteristics of typed HAV cases, we analysed the distribution by age group (0–9, 10–19, 20–29, 30–39, 40–49, 50–59, 60–69, 70–79 years) and sex. The proportions of hepatitis-related symptoms, hospitalisations, vaccinations, possible sources of infection and suspected country of origin of the virus were calculated.

### Genotyping analysis

The protocol for molecular detection and typing of the VP1/P2A genomic region of HAV (National Institute for Public Health and the Environment, the Netherlands) [15] was used for genotyping by sequencing 460 nt. Automated RNA extraction from serum samples was performed on NucliSens easyMaq, Biomerieux instrument (bioMerieux, Durham, North Carolina, United States (US)), amplification on GeneAmp 9700 thermal cycler (Applied Biosystems, Waltham, Massachusetts, US), and sequencing on Applied BioSystems 3130xl genetic analyser (Applied Biosystems). All acquired HAV sequences were submitted to the HAVNET database and compared with reference sequences, including three outbreak strains widely circulating among MSM (VRD_521_2016, RIVM-HAV16–090 and V16–25801) and reference strains for genotypes (IA, IB, IIIA) obtained from GenBank database (X75215, M20273, AY644337.1). A phylogenetic tree was inferred by using the Maximum Likelihood method based on the Tamura-Nei model with bootstrap analysis (1,000 replicates). All positions containing gaps and missing data were eliminated. The phylogenetic tree was generated by MEGA (6.0) software [16].

### Ethical statement

The planning, conduct and reporting of studies were in line with the Declaration of Helsinki, as revised in 2013 [17]. Ethics Committees of Riga East University Hospital (Reference: ZD/08–06/01–19/210) and Centre for Disease Prevention and Control of Latvia (Reference: 6.1–3/8) approved the study. HAV cases were not directly involved in this study. Only data extracted from notifiable disease surveillance systems were used. All identifiable personal information was removed for privacy protection and therefore no informed consent was required.

## Results

**Figure 1:**
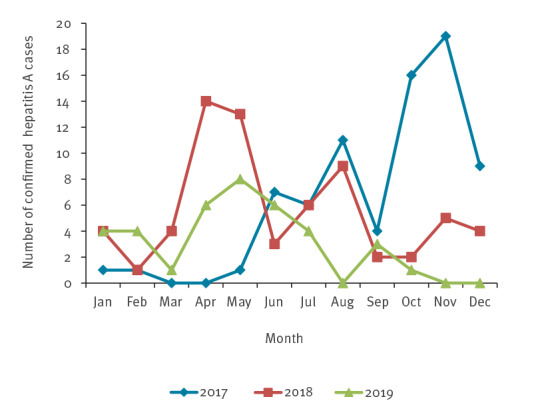
Laboratory-confirmed hepatitis A cases, Latvia, 2017–2019 (n = 179)

During 2017–19, 179 hepatitis A cases were reported in Latvia. In 2017, the first hepatitis A case was registered in January and case numbers increased from June onward, reaching a peak in November 2017. In 2018, the peak was observed in April and May, and cases decreased until December. In 2019, cases were registered throughout the whole year and the peak of cases was reached in May. All hepatitis A cases that were laboratory-confirmed by anti-HAV IgM in Latvia from 2017 to 2019 are shown (Figure 1).

All cases (n = 75 in 2017; n = 67 in 2018; n = 37 in 2019) were laboratory-confirmed for hepatitis A virus by anti-HAV IgM.

### Genotyping of hepatitis A cases

Serum samples from cases (125/179; 69.8%) were sequenced in order to determine the HAV subgenotype. The majority of samples (77/125; 61.6%) were typed as HAV subgenotype IA, while 35.2% (44/125) were HAV subgenotype IB and 3.2% (4/125) HAV subgenotype IIIA. In 2017, the majority of the typed samples were subgenotype IA (55/77), of which most belonged to one of three HAV subgenotype IA outbreak strains widely circulating among MSM during 2016/17 in Europe (VRD_521_2016, n = 29; RIVM-HAV16–090, n = 5; V16–25801, n = 2) (Table 1). However, other clusters and sporadic cases of HAV subgenotype IA infections were also detected. There were three cases with HAV subgenotype IB and one case with HAV subgenotype IIIA. In 2018, HAV subgenotype IB predominated (n = 29), while HAV subgenotype IA was detected in 20 cases and HAV subgenotype IIIA in three cases. However, three cases from cluster VRD_521_2016 and two cases from cluster RIVM-HAV16-090 were also detected in 2018. In 2019, HAV subgenotype IB also predominated with 12 cases, while HAV subgenotype IA was detected in two cases.

**Table 1:** Distribution of hepatitis A virus subgenotypes, Latvia, 2017–2019 **(**n = 125)

HAV subgenotypes	Number of cases per year			Total (n = 125)	Total %^a^
	2017 (n = 59)	2018 (n = 52)	2019 (n = 14)		
IA	55	20	2	77	61.6
IA (VRD_521_2016)^b^	29	3	0	32	41.5
IA (RIVM-HAV16–090)^b^	5	2	0	7	9.1
IA (V16–25801)^b^	2	0	0	2	2.6
IA (other cases)	19	15	2	36	46.8
IB	3	29	12	44	35.2
IIIA	1	3	0	4	3.2

HAV: hepatitis A virus.

^a^ For subgenotypes IA, IB and IIIA, total per cent represents the proportion of each subgenotype of the combined total of the three. For the IA subgenotype strains, the per cent represents the proportion of the four IA subgenotypes.

^b^ Strains associated with outbreaks of hepatitis A in Europe that have been shown to circulate among MSM during 2016/17.

### Demographics

**Figure 2:**
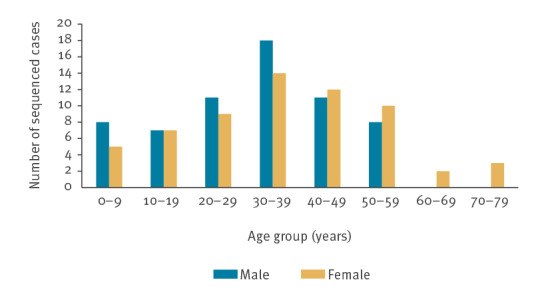
Age and sex distribution of sequenced hepatitis A cases, Latvia, 2017–2019 (n = 125)

Among the 125 cases with sequenced HAV, the age ranged from 3 to 73 years, with a median age of 33 years. Of the total, 19.2% (24/125) were children (aged 3–17 years; n = 7 in 2017; n = 14 in 2018; n = 3 in 2019) and 80.8% (101/125) were adults (aged 18–73 years; n = 52 in 2017; n = 38 in 2018, n = 11 in 2019). The male/female ratio was 63/62 (male cases: n = 32 in 2017; n = 25 in 2018; n = 6 in 2019 and female cases: n = 27 in 2017; n = 27 in 2018; n = 8 in 2019). The age and sex distribution of sequenced cases of hepatitis A are shown in Figure 2.

Of the 125 sequenced HAV cases, all reported country of birth as Latvia except two cases who reported Uzbekistan or Bulgaria. Similarly, Latvia was the country of residence for all but two cases who reported the United Kingdom (UK) or Germany.

### Epidemiological data of hepatitis A cases

Among 125 sequenced HAV cases, hepatitis A-related symptoms during the infection were known for 94.4% (118/125) cases, while 5.6% (7/125) lacked information about symptoms because clinicians did not report them. All cases were considered to be symptomatic; the prevalence of asymptomatic cases in Latvia is unknown. Of the 125 cases with hepatitis A, 89.6% (112/125) were hospitalised. Available epidemiological data about vaccination against HAV among typed cases has shown that 84.8% (106/125) were not vaccinated against HAV and 15.2% (19/125) with unknown vaccination status.

Data about possible sources of infection has shown that 28% (35/125) cases had contact with an HAV case, however 72% (90/125) possible sources of infection were unknown.

The travel history to other countries during the incubation period was known for 23.2% (29/125) cases, while 76.8% (96/125) were Latvian domestic cases. An assessment of countries of travel revealed that Austria, Bulgaria, Estonia, France, Kazakhstan, the Netherlands and Ukraine were each reported as a travel destination by one case. Two cases each reported to have visited Morocco, Russia and the UK. Three cases had visited Uzbekistan and Spain and six cases had travelled to Germany. Four of the total 125 hepatitis cases with subgenotype IIIA have the country of travel as India.

Of note, 3.2% of cases (4/125) identified themselves as MSM and all of these cases belong to cluster RIVM-016–90.

### Phylogenetic analysis

**Figure 3:**
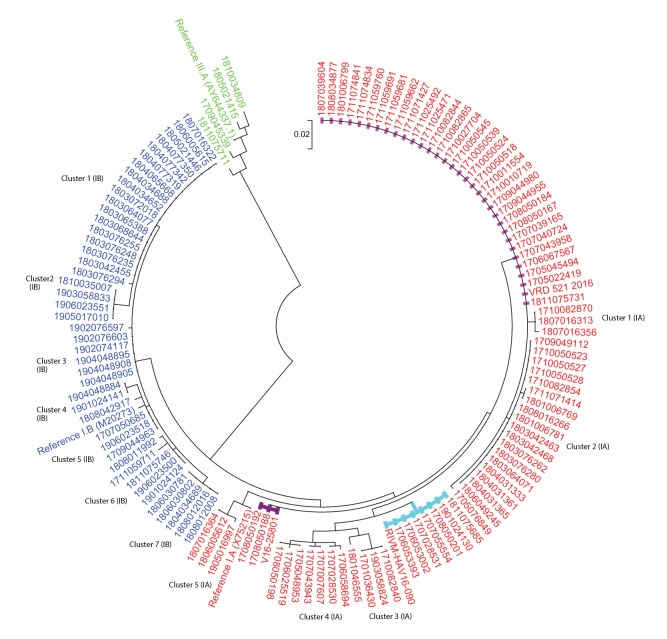
Maximum likelihood phylogenetic tree of the hepatitis A virus VP1/2A genomic region sequences from hepatitis A cases, Latvia, 2017–19 (n = 125)

Phylogenetic analysis was performed on the 125 Latvian HAV sequences with HAV subgenotypes IA, IB, IIIA (Figure 3). There were three clusters associated with outbreak strains of hepatitis A in Europe circulating among MSM during the 2016/2017 (VRD_521_2016, RIVM-HAV16–090 and V16–25801). However, other clusters were also identified.

Reference sequences for subgenotypes IA, IB and IIIA from the GeneBank database ((Reference I.A (X75215), Reference I.B (M20273) and Reference III.A (AY644337) respectively) and HAVNET database (VRD_521_2016, RIVM-HAV16-090, V16-25801) have been included for comparison with 77 HAV subgenotype IA (red text), 44 HAV subgenotype IB (blue text) and 4 HAV subgenotype IIIA (green text) sequences. The European strains circulating among men who have sex with men are marked in coloured bars: VRD_521_2016 (thin pink bars), RIVM-HAV16–090 (thick turquoise bars) and V16–25801 (thick purple bars).

HAV subgenotype IA results show that 77 subgenotype IA sequences fall into eight clusters with seven sporadic cases (Figure 3, Table 2). There were seven sequences from the RIVM-HAV16–090 cluster: four cases identified themselves as MSM, one case had contact with an HAV case and, in two cases, the possible source of infection was unknown. Based on the epidemiological data, suspected countries of origin of the virus were Germany (n = 2), Spain (n = 1), UK (n = 1), France (n = 1) and Latvia (n = 2). Cluster VRD_521_2016 consisted of 32 sequences: 11 cases had contact with HAV case, but in 21 cases, the possible source of infection was unknown. Twenty-eight hepatitis A cases were domestic; in two cases suspected county of origin of the virus was Germany, in one Spain and in one France. Cluster V16–25801 consists of 2 sequences with an unknown source of infection and from epidemiological data these cases were from Germany and Austria. Cluster 2 consists of 18 identical sequences, four had contact with an HAV case, 14 were with unknown source of infection; 16 were domestic cases, with one case each reporting travel to the Ukraine or the Netherlands. Clusters 1, 3 and 5 consist of 3 identical sequences with unknown source of infection in each cluster. Cluster 4 consists of 2 identical sequences and there were 7 sporadic cases with no identified epidemiological link.

**Table 2:** Hepatitis A virus subgenotype IA clusters, Latvia, 2017–2019 (n = 77)

Cases	Subgenotype IA clusters								
	VRD_521_2016^a^	RIVM-HAV16–090^a^	V16–25801^a^	Cluster 1	Cluster 2	Cluster 3	Cluster 4	Cluster 5	Sporadic cases
	(n = 32)	(n = 7)	(n = 2)	(n = 3)	(n = 18)	(n = 3)	(n = 2)	(n = 3)	(n = 7)
Sex									
Male	16	5	2	2	9	1	1	2	2
Female	16	2	0	1	9	2	1	1	5
Age (years)									
0–9	2	0	0	0	0	0	0	0	0
10–19	3	0	0	0	2	1	0	0	0
20–29	6	0	1	2	3	0	1	0	1
30–39	8	5	1	1	1	1	0	2	2
40–49	4	1	0	0	8	1	1	1	1
50–59	8	1	0	0	3	0	0	0	1
60–69	0	0	0	0	0	0	0	0	1
70–79	1	0	0	0	1	0	0	0	1
Possible source of infection									
Unknown	21	2	2	2	14	3	2	3	7
Contact with HAV case	11	1	0	1	4	0	0	0	0
MSM	0	4	0	0	0	0	0	0	0
Suspected country of origin of the virus									
Latvia (domestic cases)	28	2	0	3	16	0	1	1	2
Germany	2	2	1	0	0	0	0	0	1
Spain	1	1	0	0	0	0	1	0	0
United Kingdom	0	1	0	0	0	0	0	0	0
Austria	1	0	0	0	0	0	0	0	0
France	0	1	0	0	0	0	0	0	0
Estonia	0	0	1	0	0	0	0	0	0
Ukraine	0	0	0	0	1	0	0	0	0
Netherlands	0	0	0	0	1	0	0	0	0
Russia	0	0	0	0	0	1	0	0	1
Uzbekistan	0	0	0	0	0	2	0	0	1
Morocco	0	0	0	0	0	0	0	2	0
Bulgaria	0	0	0	0	0	0	0	0	1
Kazakhstan	0	0	0	0	0	0	0	0	1

HAV: hepatitis A virus; MSM: men who have sex with men.

^a^ These strains were circulating during 2016/17 among MSM populations in Europe.

HAV subgenotype IB results show that 44 sequences fall into seven clusters and three sporadic cases (Figure 3, Table 3). Cluster 1 consists of 18 identical sequences, of which 16 had a possible infection source of contact with an HAV case. Cluster 3 consists of six identical sequences with an unknown source of infection, but all cases were associated with hepatitis A outbreak in a kindergarten. Cluster 7 consists of five identical sequences, two of which had a possible infection source of contact with an HAV case; three had an unknown source. Clusters 2, 4, 5, 6 have identical sequences in each cluster with an unknown source of infection and there were 6.8% (3/77) sporadic cases with no identified epidemiological link.

**Table 3:** Hepatitis A virus subgenotype IB clusters, Latvia, 2017–2019 (n = 44)

Cases	Subgenotype IB clusters							
	Cluster 1	Cluster 2	Cluster 3	Cluster 4	Cluster 5	Cluster 6	Cluster 7	Sporadic cases
	(n = 18)	(n = 4)	(n = 6)	(n = 2)	(n = 3)	(n = 3)	(n = 5)	(n = 3)
Sex								
Male	9	1	3	0	2	3	1	2
Female	9	3	3	2	1	0	4	1
Age								
0–9	6	0	2	0	0	0	3	0
10–19	6	1	0	0	1	0	0	0
20–29	1	2	0	1	0	0	1	0
30–39	1	1	3	0	0	1	0	2
40–49	1	0	1	1	1	1	1	0
50–59	2	0	0	0	1	1	0	1
60–69	1	0	0	0	0	0	0	0
70–79	0	0	0	0	0	0	0	0
Possible source of infection								
Unknown	2	4	6^a^	2	3	3	3	3
Contact with HAV case	16	0	0	0	0	0	2	0

HAV: hepatitis A virus; UK: United Kingdom.

^a^ All cases were associated with an outbreak in a kindergarten.

HAV subgenotype IIIA results have shown that four sequences were different. All of the cases were associated with travel history to India.

## Discussion

We present an overview of hepatitis A cases from 2017 to 2019 in Latvia. In early 2017, outbreaks of hepatitis A circulating among MSM in Europe occurred, and the numbers of reported cases increased in Latvia paralleled this increase [18]. All confirmed cases of HAV from 2017–19 with no obvious source were sequenced, and we identified eight clusters of subgenotype IA and seven clusters of subgenotype IB.

Prior to this, the most recent hepatitis A outbreak in Latvia was in 2007–08. Initially, transmission occurred among PWID and other high-risk individuals, but further spread led to a community-wide increase in the number of cases [19]. This outbreak can be attributed to the large number of susceptible individuals as a result of low population immunity to hepatitis A. To control the outbreak, measures taken included contact tracing of cases, vaccination recommendations to contacts of cases, quarantine and medical observation of cases, as well as public health education through mass media and specific recommendations for prevention targeted at food handlers, schools, and the general public. Since September 2008, an increased uptake of HAV vaccination of the population has been observed. Up to 2017, few acute hepatitis A cases were reported in Latvia each year and the majority of the few cases have been associated with travel to endemic regions.

The analysis of the prevalence of HAV within a 3-year time frame revealed the presence of three HAV subgenotypes – IA, IB, IIIA – in Latvia. Of all sequenced HAV outbreak strains between 2017 and 2019, 61.6% were laboratory-confirmed HAV subgenotype IA, the most prevalent genotype worldwide [7]. The sequence similarity of 99.3% or more was found to one of the three HAV subgenotype IA outbreak strains circulating among MSM in Europe (VRD_521_2016, RIVM-HAV16–090, V16–25801) based on overlapping fragments at the VP1/2A genomic region [20-22]. Phylogenetic analysis demonstrated that these strains were also circulating in Latvia. The majority of the remaining cases were subgenotype IB (35.2%). Only four case samples were with subgenotype IIIA and all had a travel history of India.

Based on the HAV sequences and the phylogenetic tree, we speculate that increased hepatitis A cases in Latvia 2017 may be related to outbreaks of hepatitis A among MSM in Europe [20], given that the majority of cases were similar to those reported in ECDC EU/EEA countries. Other sporadic hepatitis A cases as well as those which were epidemiologically linked were domestic cases. From three MSM-associated HAV clusters (VRD_521_2016; RIVM-HAV16–090; and V16–25801), we identified 23 male and 18 female cases. These clusters were probably either imported by MSM and transmitted to others following contact with a HAV case or via travel.

Previous studies underscore that travel to countries with high or intermediate HAV endemicity is a risk factor in residents of countries with low HAV endemicity, and continues to be a major risk factor for HAV infection in the EU/EEA [1]. Of the EU/EEA countries reporting HAV data, 27.8% of reported hepatitis A cases were travel-associated [23]. Our findings show that 23.2% of hepatitis A cases were also associated with travel during the HAV incubation period. The first cases of hepatitis A reported in Latvia during 2017 were associated with travel to Germany, Spain, Austria and the UK.

The global incidence of hepatitis A has declined over the past years, primarily because of hepatitis A immunisation. However, our data from Latvia show that 84.8% of the cases between 2017–19 were not vaccinated and 15.2% had an unknown vaccination status. Currently, the hepatitis A vaccine is not included in the national immunisation calendar in Latvia. Vaccination is only recommended for citizens who travel to countries or areas where HAV is endemic or have with low or intermediate levels of infection. However, more often than not, these travel recommendations are ignored.

Additional hepatitis A prevention strategies including detailed recommendations for different target groups (food-handlers, childcare establishments, and the general public) have been developed and distributed to different institutions at national and local levels to increase the knowledge in the field of hepatitis A among the population and healthcare workers [24]. 

### Limitations

Our study has some limitations. The main limitation of the HAV surveillance in Latvia was the challenge to understand the possible source of infection. The most common reported source of infection was a household or other close contact with an infected person. Other potential sources of infection include contact with a risk group, e.g. MSM, travel to HAV-endemic countries, and PWID. Contaminated food and water are an infrequent source of infection, although they have been associated with outbreaks [25]. Our results shown that the main sources of infection that can be reported in Latvia are cases of contact with the HAV case (28%) and HAV cases associated with travel (23.2%). Food-borne source of infection mostly had never been reported because of the long incubation period of HAV infection and this is a major issue of public health response.

Also, our data lacked information about the sexual habits of cases, only four cases identified themselves as MSM, although these cases were also associated with travel. 

## Conclusion

We report the first detailed comprehensive molecular epidemiological study of HAV in Latvia, which highlights genetic diversity of HAV circulating in the country. The combination of diagnostic methods, methods of molecular biology and epidemiological data allow public health to identify clusters, establish links with other outbreaks and compare Latvian strains with other strains. This approach is helpful in understanding the epidemiological process of hepatitis A.
